# Supplementary material for: MetaRibo-Seq measures translation in microbiomes
Source: Nat Commun. 2020 Jun 29;11:3268. doi: 10.1038/s41467-020-17081-z (PMC7324362; doi:10.1038/s41467-020-17081-z)
Supplement: Supplementary file 10 — Supplementary Data 7 [file 41467_2020_17081_MOESM10_ESM.zip › File2/Confidence_High_Taxonomy/293487_out.krona.html]

Javascript must be enabled to view this page.

members
magnitude
magnitudeUnassigned
count
unassigned
taxon
rank

293487\_out

33

superkingdom
2
33

phylum
1239
33

186801
class
33

order
186802
33

family

SRS104975\_contig\_number\_501
1
33
186803

genus
1407607
7

7

SRS018427\_contig\_number\_1368SRS019068\_contig\_number\_2136SRS022071\_contig\_number\_31923SRS050941\_contig\_number\_13035SRS104400\_contig\_number\_36679SRS1055043\_contig\_number\_9846SRS144537\_contig\_number\_5283
1150298
species

genus

SRS015578\_contig\_number\_3967SRS063127\_contig\_number\_1381SRS104636\_contig\_number\_28848SRS143780\_contig\_number\_contig-100\_1023.71979SRS143991\_contig\_number\_contig-100\_2916.95154SRS147139\_contig\_number\_9973SRS147346\_contig\_number\_35139SRS971275\_contig\_number\_30803
572511
8
23

species
2292990

SRS064276\_contig\_number\_7922
1

1262757
species

SRS015217\_contig\_number\_22908SRS020869\_contig\_number\_27176SRS077454\_contig\_number\_16225SRS100021\_contig\_number\_3116SRS144183\_contig\_number\_36181
5


SRS148721\_contig\_number\_17459
species
2292962
1

33039
species

SRS051882\_contig\_number\_17664
1

1
1896976
species

SRS077502\_contig\_number\_18688


SRS077392\_contig\_number\_31807SRS097889\_contig\_number\_36743SRS1041036\_contig\_number\_2772SRS1041145\_contig\_number\_34135SRS105153\_contig\_number\_26244SRS146813\_contig\_number\_2920
species
2292976
6


SRS098644\_contig\_number\_27295
genus
841
2
1

1
2049040
species

SRS100021\_contig\_number\_7916
